# Passive Sensing of Prediction of Moment-To-Moment Depressed Mood among Undergraduates with Clinical Levels of Depression Sample Using Smartphones

**DOI:** 10.3390/s20123572

**Published:** 2020-06-24

**Authors:** Nicholas C. Jacobson, Yeon Joo Chung

**Affiliations:** 1 Center for Technology and Behavioral Health, Geisel School of Medicine, Dartmouth College, Lebanon, NH 03766, USA; chungisab@gmail.com; 2 Department of Biomedical Data Science, Geisel School of Medicine, Dartmouth College, Lebanon, NH 03766, USA; 3 Department of Psychiatry, Geisel School of Medicine, Dartmouth College, Lebanon, NH 03766, USA

**Keywords:** major depressive disorder, digital phenotyping, digital biomarkers, machine learning, ecological momentary assessment

## Abstract

Prior research has recently shown that passively collected sensor data collected within the contexts of persons daily lives via smartphones and wearable sensors can distinguish those with major depressive disorder (MDD) from controls, predict MDD severity, and predict changes in MDD severity across days and weeks. Nevertheless, very little research has examined predicting depressed mood within a day, which is essential given the large amount of variation occurring within days. The current study utilized passively collected sensor data collected from a smartphone application to future depressed mood from hour-to-hour in an ecological momentary assessment study in a sample reporting clinical levels of depression (*N* = 31). Using a combination of nomothetic and idiographically-weighted machine learning models, the results suggest that depressed mood can be accurately predicted from hour to hour with an average correlation between out of sample predicted depressed mood levels and observed depressed mood of 0.587, CI [0.552, 0.621]. This suggests that passively collected smartphone data can accurately predict future depressed mood among a sample reporting clinical levels of depression. If replicated in other samples, this modeling framework may allow just-in-time adaptive interventions to treat depression as it changes in the context of daily life.

## 1. Introduction

As students leave their homes and enter college, they have to cope with numerous adjustments that follow from their transition to college, including academic, social, personal or emotional and institutional attachment [1]. Relative to non-college peers, college students are more likely to suffer from symptoms of depression but less likely to seek treatment [2]. Major depressive episodes are most prevalent between 18 and 25 years old, [3] which coincides with the period in which most students enter college. Studies have found that an increasing number of college students have experienced “severe psychological problems”, including suicides and crisis management, in recent years [4] and that mental health issues are affecting up to eight times as many college students as it did in the Great Depression era [5]. This implies that current undergraduate students are more prone to the fatal conditions associated with major depressive disorder (MDD) such as cardiovascular disease, diabetes mellitus [6] and notably death by suicide [7]. In addition to the detrimental health risks, depression is also associated with lower grade point averages, dropping more courses and missing more classes, exams, and assignments as well as social activities [7]. It is thus critical to find a method that better predicts depression and facilitates diagnosis and intervention, ideally by using passively collected data through a technology owned by a staggering 85% of American undergraduates [8].

A variety of studies have attempted to predict depression severity from smartphones and wearable sensors during one cross-sectional timepoint [9,10,11,12,13,14,15,16,17]. In almost all studies, prior research has shown a significant relationship between predicted depressive symptoms severity using passive sensor data and actual symptom severity, with associations ranging from a correlation as high as 0.55 on a specific feature, 0.74 area under the curve receiver operator characteristic curve (AUC), F_1_ score of 0.85 and accuracy ranging from 86.5 to 89.4% (73–89% sensitivity and 91–97% specificity). 

Other research has focused on predicting changes in depression severity across varying numbers of weeks, including across ten weeks via mobile phone location sensor data [14], eight weeks via smartphone mobile sensing and support [18], twelve weeks via smartphone data [19,20], eight weeks via a smartphone-based monitoring system [21], twelve weeks in bipolar patients via smartphone behavior and activity monitoring [22], across twelve weeks in bipolar patients via smartphone sensors, specifically inertial sensors and GPS traces [23], thirteen weeks via smartphone sensors and wearable sensors [24], nine days via smartphone sensors [25], one week via geographic location data [26], two weeks via smartphone-based multi-modal sensing [27], three and six week periods via mobile phone sensors and location data [11], eight weeks via smartphone keystrokes [28], and a few weeks via a smartphone-based system for gathering data about social and sleep behaviors [29]. Such data has tended to find a significant relationship between predicted depressive symptoms severity using passive sensor data and actual symptom severity, with associations ranging from an area of 0.74 under the AUC curve, F_1_ score ranging from 0.77 to 0.85 and accuracy ranging from 59.1 to 84.9% (62.3–97% sensitivity, 47.3–87.2% specificity). Nevertheless, there is sparse research looking at depression within short-time fluctuations in the context of daily life. 

In contrast to the many studies that have examined passive sensor data in predicting depression severity across weeks to months, few studies have examined predicting depressed mood across hours or days [13,30,31]. Importantly, shifts in MDD symptoms occur rapidly with substantial fluctuations occurring over the course of a day or even hour-to-hour [32,33,34,35]. Consequently, it is essential to predict MDD symptoms across intervals as short as hours. Canzian and Musolesi (2015) predicted daily mood by examining daily location data from smartphone sensors, finding that geolocation data was correlated with depression severity on a day-to-day basis and that geolocation data could be used to predict dichotomous depression severity from 1 to 14 days later. Pratap et al. (2019) predicted daily dichotomized depressive severity within individuals and variance explained across individuals, finding that idiographic models could predict the depression scores in a sample of persons at clinical levels of depression. Lastly, Burns et al. (2011) predicted dichotomized depression 5 times per day using smartphones sensors. Of these studies, two of three studies found strong agreement between predicted and observed daily depression outcomes (although this depended upon the analytic approach) [30,36], whereas one study found that they were unable to significantly predict depressed mood within the day in a small sample [31].

Note that there are some substantial limitations to prior studies that predicted depression in intensively sampled periods which should be addressed. Firstly, each of the prior studies dichotomized their depression outcomes when examining their primary outcomes, rather than looking at whether depressed mood could be predicted across a continuum [13,30,31]. Secondly, two of the three studies did not examine depressed mood within days, which neglects the substantial mood changes in MDD across a single day [32], rapid mood fluctuations on an hourly basis [33,35,36], and a great deal of variation in depressed mood not being stable across more than several hours [37]. Consequently, most prior research is unable to adequately translate to inform just-in-time adaptive interventions (JITAI) [38,39], as this research might miss important times in which persons might be experiencing depressed mood fluctuations. Moreover, the one study that did examine intraindividual changes in depressed mood across the day had a small sample size (*N* = 11), and also added an intervention [31]. Consequently, research is needed to examine the context of intraindividual shifts within days across a continuum of depressed mood during its naturalistic course. 

Of the current studies, research has increasingly highlighted the importance of considering large interindividual differences in MDD which may diminish model generalization to other persons [13,40,41,42,43]. Although such research has favored idiographic modeling techniques over between-person work [44], this shift as an either idiographic or nomothetic methods as dichotomous decisions is a contrived dichotomy [45]. In contrast, idiographic and nomothetic models might be balanced by weighting an individual heavily, but still being informed by the general context of others, which has been proposed in prior reviews [46]. 

The current work integrates several types of passive sensing data including integrating several different types of signals including physiology, movement, location, light, and phone calls to predict future changes in depressed mood in a sample of persons at clinical levels of depression. Importantly, this is the first study to integrate both passive mobile sensor data and physiology [47]. We hypothesized that we could significantly positively predict depressed mood across time in a sample of undergraduates with MDD.

## 2. Materials and Methods

### 2.1. Participants

Participants (*N* = 31, 64.52% female, *M* age = 19.129, age range 18–27, 67.74% Caucasian, 6.45% African American, 3.22% Hispanic/Latino, 16.12% Asian American, and 6.45% Other) were recruited to participate in a study on predicting mood. Participants were recruited from an undergraduate participant pool. To qualify for the present study, participants mood scores needed to exhibit significant variation in their mood across time based on a previously utilized interquartile range [13,48], note this requirement was put in place to be conservative as the predictive performance of these models would likely be much higher in the absence of substantial change. The depression severity of the sample was as follows: 6.45% [2] of the sample met moderate depression severity, 38.7% [12] of the sample met severe depression severity, and 54.8% [17] met very severe depression severity [49]. Thus, the majority of persons included in the current sample were very severely depressed. All participants gave their written informed consent to participate in the study and Pennsylvania State University approved the study.

### 2.2. Protocol

Participants were recruited from a subject pool in a large university in the Northeast. Participants were recruited via an online portal and were able to enroll in the study at this point, and they were granted participation credit for participating in the current study. Participating in this course credit counted towards their introductory-level psychology course. To participate in the current study, participants were required to own an Android based phone. Participants then attended an introductory session where they were asked to install the “Mood Triggers” application on their phones. Mood Triggers is an application that collects ecological momentary assessment data and passive sensing data and gives users feedback about which features most strongly predict their anxiety and depressed mood. At this point participants completed baseline measures (i.e., the Depression Anxiety and Stress Scale). Participants were also asked to input the hours they stated that they would be awake over the following seven days, by inputting their bedtimes and wakeup times. Following this point, participants were prompted to rate their depressed mood once per hour and a heart rate assessment for the times that they indicated that they would be awake (note that participants also completed other measures outside the bounds of the current study). During study enrollment passive sensor data was also passively collected throughout the study period. Participants then returned to the laboratory where their data was downloaded from their phone approximately eight days later.

### 2.3. Measures

#### 2.3.1. Baseline Depression Severity

*Depression Anxiety and Stress—Depression Scale.* This scale was used to measure the magnitude of depression based on responses from a 14-item self-administered questionnaire. The scale assesses dysphoria, hopelessness, devaluation of life, self-deprecation, lack of interest/involvement, anhedonia, and inertia, which are all core symptoms of MDD [50]. Examples of items on the scale includes “I couldn’t seem to experience any positive feeling at all” and “I just couldn’t seem to get going”. Participants rate on a 4-point scale the extent to which such a statement applied to them over the past week. The maximum and minimum possible scores are 42 and zero, respectively. Higher scores indicate greater depression. Internal consistency reliability has been demonstrated with Cronbach’s alpha values of 0.97 for the total scale and 0.96 for the Depression scale [51]. Test-retest reliability has been demonstrated by a correlation of 0.713 between two administrations of the Depression scale across two weeks [52]. Discriminant validity has been demonstrated by studies showing that this measure is capable of discriminating depression from other disorders such as panic disorder and generalized anxiety disorder [52], performing best in the mild-moderate severity [53]. Convergent validity of this instrument has also been demonstrated by a strong correlation as high as 0.75 with various measures of depression [52].

#### 2.3.2. Dynamic Depressed Mood

Dynamic depressed mood was measured using the “sad” and “lonely” items of the Positive and Negative Affect Schedule Expanded (PANAS-X). The two items assess to what extent participants felt those two negative emotions, which are both core constructs to MDD. Participants were asked, once per hour for each hour they were awake, to rate on a 100-point scale the extent to which they felt (1) sad and (2) lonely “right now” (at the time of data collection). Ratings were obtained every hour using the *Moment* instructions of the PANAS-X scale, as the current study aims to predict hourly depressed mood. The maximum score of 100 indicates that the participant feels a certain emotion “extremely”, and the minimum score of zero indicates “not at all”. Higher scores on sadness/loneliness indicate greater depressed mood. Prior research suggests loneliness is strongly linked to major depressive disorder [54,55,56,57]. In addition to loneliness, sadness is another core construct to MDD. Reis (1989) has identified sadness and loneliness as two key measures of depressed affect in a sample of young adolescent mothers where 67% were depressed, with the “sad” and “lonely” items reporting respective correlations of 0.80 and 0.65 in a Varimax rotated matrix of CES-D Depression scores [58]. In particular, it may be worthy to note that the two items were more strongly associated with depression than the “depressed” item itself (which had a coefficient of 0.49), suggesting that self-reported sadness and loneliness may be strong predictors of depression (thus corroborating/justifying the current study’s use of the “sad” and “lonely” items to assess dynamic depressed mood). Furthermore, another study demonstrated strong convergent validity (*r* = 0.66 to 0.67) between sadness and loneliness [59]. 

#### 2.3.3. Passive Sensor Data

A number of features were passively collected from participants including: (1) direct location based information: (1a) GPS coordinates (latitude, longitude), (1b) location accuracy, (1c) location speed, and (1d) whether the location-based information was based on GPS or WiFi; (2) location type based on the Google Places location type (e.g., University, gym, bar, church); (3) local weather information, including (3a) temperature, (3b) humidity, (3c) precipitation, (2) light level, (3) heart rate information: (3a) average heart rate and (3b) heart rate variability; and (4) outgoing phone calls. 

The sensing data was indexed once per hour on the hour. This decision was adopted due to the ecological momentary assessment design, and in order to prevent excessive battery drain. In particular, using the GPS location more frequently can cause considerable battery drain. The app defaulted to use GPS location when the user did not have the location services disabled. However, when the GPS location was disabled, the app collected location-based information from WiFi (and we used this as a feature as noted above). The type of location was then processed to codify whether the nearest location based on Google Places as well as the local weather information as indexed through the National Weather Service API. To keep data collection consistent, we summed the number of outgoing phone calls per hour.

Note that heart rate was measured by asking subjects to press their finger against the rear camera for 30 s, and the application measured the rapid changes of color in their finger over the 30 s period. The application noted the timing of the varying degrees of redness in the image, with high redness values corresponding to a pulse. Average heart rate was based on the average of the times between beats, whereas heart rate variability reflected the root mean square of successive differences of these beats. Results have shown that these methods have high convergence with traditional measures (*r* = 0.98–1.00 with heart rate, and *r* = 0.90–0.97 with Root Mean Square of Successive Difference [RMSSD]) [60].

### 2.4. Planned Analysis

All modeling was accomplished via machine learning algorithms. The goal of the modeling strategy was to try to utilize the past 24 h of sensor data to predict the next hour chance of depression symptom severity based on the passive data from the next hour. All models were evaluated based on out of sample model predictions. Due to the nature of time-series data it is important that the data training and validation proceeds in a way that does not artificially reverse temporal directionality (i.e., using a current sensor to predict an outcome that occurred in the past). Consequently, models need to be trained based on only on data from data that occurred within the past to predict present moment data. Consequently, we chose 24-h rolling windows to predict the outcome. We chose this strategy rather than all utilizing all previously observed data as this might affect the model precision over time; whereby the precision of the model might change as a function of the length of time in the study. As we wanted to optimize whether this procedure would be valid if trained on data from one day and generalized to the hour following this period, we chose not to utilize all prior data, but to use this windowed approach. 

**Figure 1:**
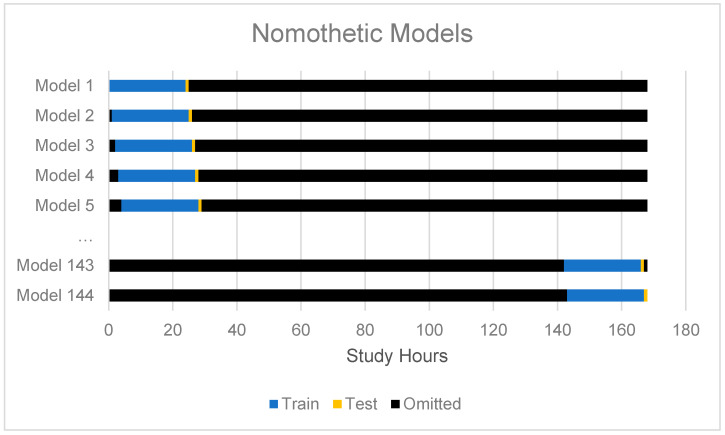
This figure describes the strategy for cross-validation for group-based nomothetic models. Note that the only the past 24 h periods were utilized to train the next hour, and a separate model was trained for each hour.

Modeling proceeded in two primary phases: (1) nomothetic modeling, and (2) idiographically-weighted modeling. First, we modeled the intraindividual variability using a nomothetic extreme gradient boosting algorithm (XGBoost) using the passive sensor data to arrive at common model predictions of intraindividual variability. These predictions were not directly of interest, but were only utilized as secondary features for the idiographically-weighted modeling. See Figure 1 for the training and cross-validation scheme for the nomothetic model of intraindividual variation. In this second phase, all features and the nomothetic predictions made for each person were modeled using the random forest models. Here we chose random forest models over extreme gradient boosting because of the computational efficiency of random forests compared to extreme gradient boosting, where we trained a new model to make idiographic predictions for each person (i.e., 31 persons × 144 out of sample predictions = 4464 idiographically-weighted models). Note that a grid search was used to optimize the number of trees to grow (based on a sequence of length three between the number of predictors) and to select the split tree rule based on variance or extremely randomized trees [61,62]. Models were optimized based on their performance in the training set and internal cross-validation set (not the test set).

**Figure 2:**
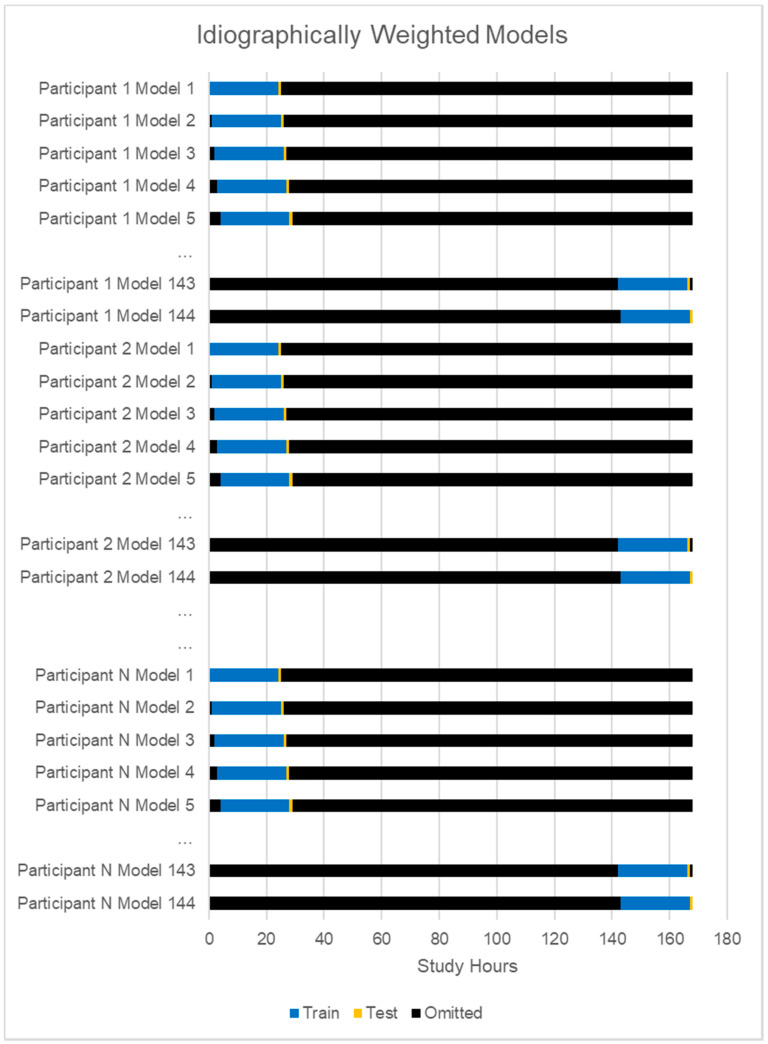
This figure describes the strategy for cross-validation for idiographic models.

A very important step in this modeling approach was to again utilize the fully sample data, but to weight a given persons’ predictions much more heavily where an individual was weighted at 1, and all other persons in the model were weighted 0.2, such that the patterns in model training strongly favored a person’s idiographic patterns. See Figure 2 for the idiographically-weighted cross-validation scheme. All presented results are based on multiple imputed data.

We also ran sensitivity analyses. Based on comments from an anonymous reviewer, we also tested whether the model results generalized across racial groups using a multilevel model (outcome_i,t_ ~ β_0_ + β_1_*prediction_i,t_ + β_2_*race + β_3_*prediction_i,t_ *race_i_ + u_i_), where β_3_ reflects the predictive performance between the prediction and the outcome as moderated by race for individual *i* at time *t*. Based on a second anonymous reviewer, we also tested whether the relationship between prediction and outcome was significant when controlling for the lagged time outcome (in case the model was just carrying forward prior timepoints using the following model (outcome_i,t_ ~ prediction_i,t_ β_0_ + β_1_*prediction_i,t_ + β_2_*laggedoutcome_i-1,t_ + u_i_).These models tested [1] whether the predictive performance varied significantly across racial groups and [2] whether the models were just learning to carry forward the last observed data point. 

## 3. Results

### 3.1. Compliance

The average participant completed a total of 51.74 prompts (range 32–93). There was a total of 1982 data points on hourly depressed mood in the current study.

### 3.2. Sensing Data

**Figure 3:**
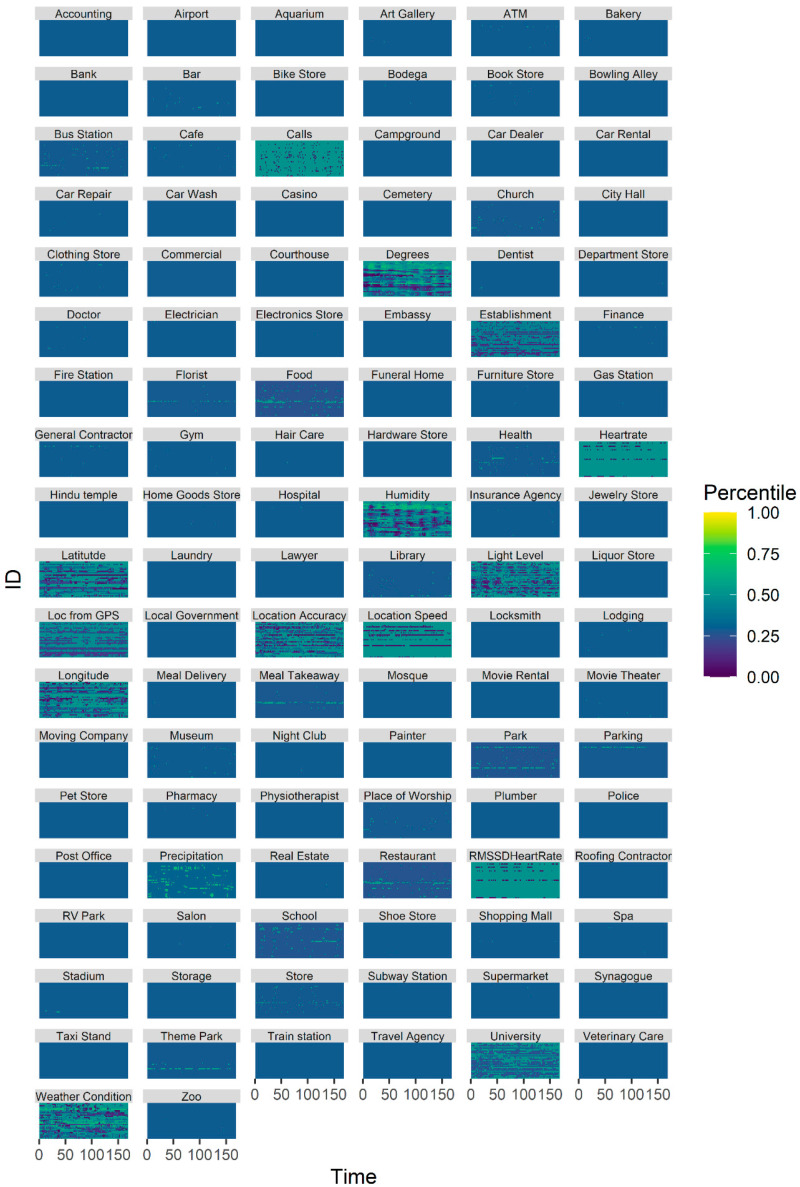
This plot depicts the percentile of the sensor values for each of the sensors across time for each subject.

The sensing data evidenced both interindividual and intraindividual variability (see Figure 3). 

### 3.3. Predicting Depressed Mood

The results suggested that the predicted depressed mood scores were highly correlated with the observed depressed mood scores from the models (*r* = 0.587, 95% CI [0.552, 0.621]), see Figure 3. Note that this includes both intraindividual variability and interindividual variability. 

### 3.4. Idiographic Predictions

**Figure 4:**
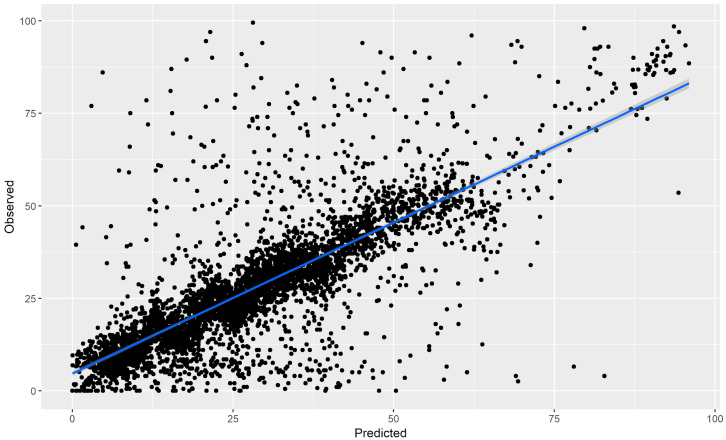
This plot depicts the predicted and observed values for the hourly depressed mood for the entire sample. For plotting, the average of the multiply imputed data points was computed when there was missing data.

**Figure 5:**
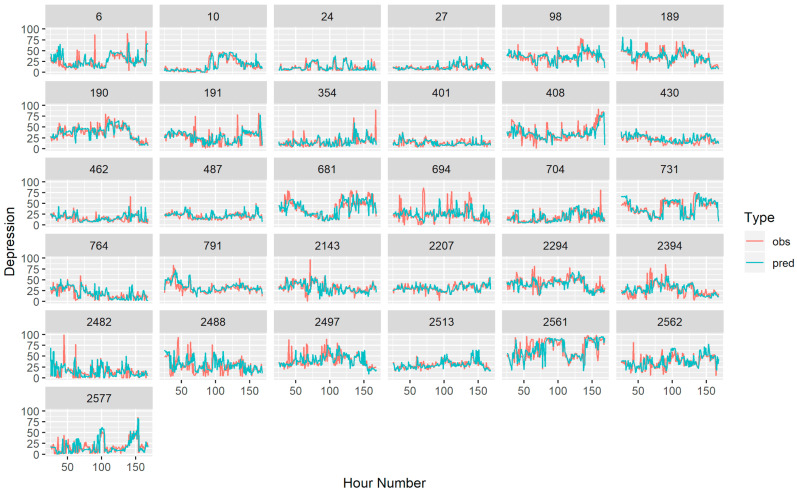
This figure depicts the hourly depressed mood for each subject. Here the red lines represent observed depressed mood points (and the average of the multiply imputed data points when there was missing data). The blue lines depict the predicted depressed mood based on the idiographically-weighted models.

**Figure 6:**
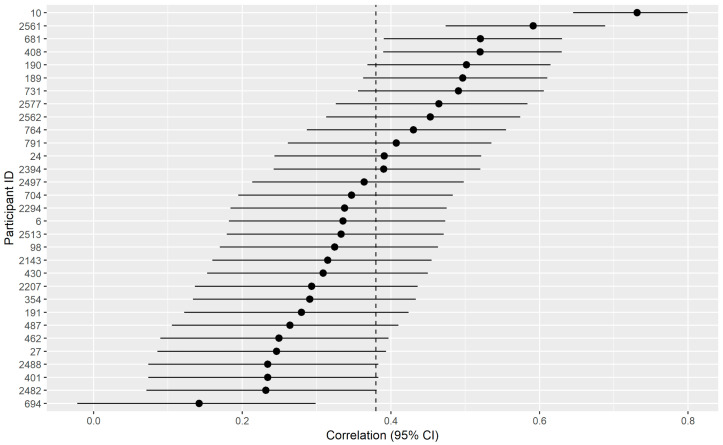
This model describes the correlation coefficient between the predicted and observed changes in the hourly mood for each participant. The vertical dotted line indicates the average of the correlations coefficient (as correlations do not scale linearly, this was done by computing r-to-Fisher’s Z transformations, taking the average, and then Fisher’s Z-to-r transformations). The dots represent the individual correlations, and the horizontal lines represent the confidence intervals around the correlation coefficient.

In addition to being interested in predicting depressed mood across all conditions, we were also interested in predicting only intraindividual variability within each person (see Figure 4). The results suggested that there was significant intraindividual variability predicted with an average correlation of 0.376, 95% CI [0.226, 0.508]. The results also suggested that the models significantly predicted intraindividual variability for all but one person (see Figure 5 and Figure 6), and even for this person the correlation coefficient bordered on the edge of significance *r* = 0.18 CI [−0.022, 0.299]. On the other hand, the largest correlation was *r* = 0.731, 95% CI [0.645, 0.799].

### 3.5. Follow-up Sensitivity Analyses

In our first sensitivity analysis, we checked whether race significantly moderated the predictive accuracy. The results suggested that there was no significant interaction between race and the model prediction, suggesting that race did not significantly moderate predictive performance (*F*(4, 3693.7) = 0.754, *p* = 0.555), which suggests that there was no significant impact of race on the model’s predictive performance. In our second sensitivity analysis, the model predictions continued to predict depression when controlling for the prior lagged depression (i.e., β_1_ = 0.543, SE = 0.016, t (4353) = 34.877, *p* < 0.001), suggesting that the main findings above were not simply a result of the model carrying the last outcome forward in time. 

## 4. Discussion

The current study examined the ability to utilize idiographically-weighted machine learning models and passive sensor data to predict the hourly mood across a week a cohort of undergraduate participants at or above moderate to very severe levels of depression severity. The results suggested that the correlation between observed and predicted hourly mood was significant, positive, and strong (*r* = 0.587). Given that the data were trained using future predictions in 24-h intervals, this suggests that a single day of passive sensor data can strongly and accurately predict the hourly depressed mood. 

Notably, another goal of the current study was to examine how well these idiographically weighted models generalized to each person by only examining the person-specific relationships of the predicted and observed mood in the current study. The results suggested that there was a moderate strength in the correlation between the idiographic predictions of depressed mood for each person (*r* = 0.376). Nevertheless, there were also significant differences in the strength of the effect across persons. The relationship between predicted and observed depressed moods for each participant was significant for all but one person (i.e., 97% of the sample). Even for this participant, the correlation was still positive, and the lower confidence interval was close to 0. On the other end, the idiographic correlations between predicted and observed depression severity were quite high for other participants (with the strongest idiographic correlation at *r* = 0.731). Given that race did not moderate the predictive performance, future research should examine between-person models which may help to explain varying levels of predictive performance across persons. In particular, anxiety is related to digital biomarkers [63,64] and is highly interrelated to depression across time [65,66,67,68]. Additionally, future work should also consider potential digital phenotypes of observed environmental stressors, given their potential for societal impacts on mood [69,70].

The current research extends prior research in several notable ways (see Table 1). Although one very small study showed that depressed mood could not be accurately predicted when operationalized as a dichotomous variable within the day (i.e., either depressed or not depressed) [31], the current research suggests that depressed mood can be significantly positively predicted within hour-to-hour time windows when operationalized continuously. This corroborates the general pattern of findings across predicting dichotomous depressed mood in longer time scales (i.e., daily mood instead of hourly mood), although again this suggests that depressed mood can be also be accurately predicted when operationalized across a continuum, rather than just considered as dichotomous [13,30]. 

Taken together, these results suggest that passive sensor data are best capable of detecting fluctuations in depression severity not when depressed mood is considered as a dichotomous variable, but rather when measured continuously within shorter hour-to-hour time windows. Therefore, the current study is able to not only predict depression but also estimate with high accuracy hourly mood changes in undergraduate students with varying levels of depression severity.

The current study has many notable strengths. Firstly, this study demonstrated the ability to predict hour to hour depression using digital phenotyping methods in the present sample, which is the shortest interval tested in research to date. The ability to predict depression in these short intervals may be particularly important in translating these assessments to just-in-time adaptive interventions, as it might facilitate timely intervention with accuracy down to the hour. Secondly, this study is the first known study to combine physiological assessments (i.e., heart rate and heart rate variability using photoplethysmographic signals) with digital phenotyping of depression [47]. Thirdly, this study demonstrates that, beyond predicting a dichotomous depression outcome, digital phenotyping of smartphone sensor data was capable of detecting continuous fluctuations in depression severity in the present sample.

Nevertheless, it is important to also discuss the limitations of the current data collection. Firstly, although the majority of the sample fell within the very severe range of depression range, this was based exclusively on a self-report assessment. Consequently, future research is needed to determine whether this research generalizes to a sample of persons meeting MDD criteria based on a clinical interview. Moreover, although this research demonstrated that digital phenotyping could be used to predict moment-to-moment depression in a sample of undergraduate students, it remains an important and unknown question whether the current research generalizes to persons with types of samples (e.g., persons with MDD in outpatient settings, older adults). As such, future work should be devoted to determining whether the same methods can generalize to those with MDD. Although the current research examined depressive mood features that appear to be particularly salient in MDD (i.e., sadness and loneliness), the current research only examined two depressive mood components. Future research should address whether the current research extends to a range of depressive mood constructs which could fluctuate intensively in daily life outside the bounds of depressed mood itself (e.g., lethargy, behavioral activation). The current research should also be extended to future settings outside the context of a mood tracking applications.

Taken together, the current research continues to build on prior research to suggest that digital phenotyping using passive smartphone sensor data may be a powerful tool in capturing moment-to-moment shifts in depressive moods among those at severe to very severe levels of MDD. Future work should examine the potential clinical utility of the present findings by using such types of digital phenotyping to inform both adaptive interventions and just-in-time adaptive interventions. With the promise of current assessments in detecting fine-grained changes in depressed mood across time, digital phenotyping and machine learning may facilitate a new area of time-specific precision medicine to enhance care scalable among those with MDD. 

## Figures and Tables

**Table 1:** This table describes prior studies that have used predictive modeling to predict future depressive symptoms and have reported predictive outcome metrics among general samples or samples high in clinical depression, but not meeting criteria for other primary diagnoses (e.g., bipolar disorder).

Study	Timescale	Design Summary	Result
[18]	8 weeks	smartphone mobile sensing and support	59.1–60.1% accuracy, 62.3–72.5% sensitivity, 47.3–60.8% specificity
[24]	13 weeks	smartphone sensors and wearable sensors	R^2^ = 0.44, F1 = 0.77
[11]	3–6 weeks	mobile phone sensors and location data	AUC = 0.88
[30]	1–14 days later	location data from smartphone sensors	71–74%-sensitivity, 78–80% specificity
[13]	daily	smartphone sensors	median area underthe curve [AUC] > 0.50) for 80.6% of persons
Current Study	Hourly	smartphone sensors	*r* = 0.587 across persons, *r* = 0.376 within persons
